# Living in two worlds: Evolutionary mechanisms act differently in the native and introduced ranges of an invasive plant

**DOI:** 10.1002/ece3.3869

**Published:** 2018-01-29

**Authors:** Wen‐Yong Guo, Carla Lambertini, Petr Pyšek, Laura A. Meyerson, Hans Brix

**Affiliations:** ^1^ Department of Invasion Ecology Institute of Botany The Czech Academy of Sciences Průhonice Czech Republic; ^2^ Department of Bioscience Aarhus University Aarhus C Denmark; ^3^ Department of Agricultural Science University of Bologna Bologna Italy; ^4^ Department of Ecology Faculty of Science Charles University Prague Czech Republic; ^5^ Natural Resources Science The University of Rhode Island Kingston RI USA

**Keywords:** biological invasions, common reed, evolution, human activities, isolation by distance, isolation by environment, *landscape genetics*, *Phragmites*, spatial genetic structure

## Abstract

Identifying the factors that influence spatial genetic structure among populations can provide insights into the evolution of invasive plants. In this study, we used the common reed (*Phragmites australis*), a grass native in Europe and invading North America, to examine the relative importance of geographic, environmental (represented by climate here), and human effects on population genetic structure and its changes during invasion. We collected samples of *P. australis* from both the invaded North American and native European ranges and used molecular markers to investigate the population genetic structure within and between ranges. We used path analysis to identify the contributions of each of the three factors—geographic, environmental, and human‐related—to the formation of spatial genetic patterns. Genetic differentiation was observed between the introduced and native populations, and their genetic structure in the native and introduced ranges was different. There were strong effects of geography and environment on the genetic structure of populations in the native range, but the human‐related factors manifested through colonization of anthropogenic habitats in the introduced range counteracted the effects of environment. The between‐range genetic differences among populations were mainly explained by the heterogeneous environment between the ranges, with the coefficient 2.6 times higher for the environment than that explained by the geographic distance. Human activities were the primary contributor to the genetic structure of the introduced populations. The significant environmental divergence between ranges and the strong contribution of human activities to the genetic structure in the introduced range suggest that invasive populations of *P. australis* have evolved to adapt to a different climate and to human‐made habitats in North America.

## 1 INTRODUCTION

While species invasions can have severe negative effects on the environment, economy, and human well‐being (e.g., Pyšek & Richardson, 2010; Vilà et al., 2011), they also represent opportunity to investigate eco‐evolutionary and biogeographic phenomena, such as range expansion, natural selection, and rapid contemporary evolution (e.g., Cronin, Bhattarai, Allen, & Meyerson, 2015; Guo, Lambertini, Nguyen, Li, & Brix, 2014; Hierro, Maron, & Callaway, 2005; Lin, Klinkhamer, & Vrieling, 2015). This is possible due to long‐term isolation of source native and invading populations and, in the majority of cases, different environmental conditions in the new range (Colautti & Lau, 2015; Guo et al., 2016; Hierro et al., 2005; Kueffer, Pyšek, & Richardson, 2013). When invasive species are establishing in the new range, they often suffer founder effects, bottlenecks, and eventually genetic drift as a result of finite numbers of individuals in the new colony (Bossdorf et al., 2005; Dlugosch & Parker, 2008; Sax et al., 2007), self‐compatibility (Petanidou et al., 2012; Zhu, Barrett, Zhang, & Liao, 2017), or asexual reproduction (Groeneveld, Belzile, & Lavoie, 2014; Hollingsworth & Bailey, 2000). These processes can considerably decrease the genetic variation and change the allelic frequencies compared to the populations in the regions of origin (Bouton, 2000; Taylor & McPhail, 1999). On the other hand, gene flow, either via multiple introductions from the original range, propagule dispersal (gametes/individuals) in the new range, outcrossing, and novel genetic admixtures, can mitigate founder effects by increasing genetic diversity and facilitate adaptation in the new range (e.g., Kolbe et al., 2004; Meyerson & Cronin, 2013).

Landscape factors, such as geographic corridors and barriers, and other environmental conditions, have a strong influence on gene flow as they create, or constrain, dispersal and establishment opportunities, and shape the spatial genetic variation accordingly. Two mechanisms, isolation by distance (IBD, Jenkins et al., 2010; Wright, 1943) and isolation by environment (IBE, Wang & Bradburd, 2014) have been proposed to explain the spatial variation patterns of plants (Wang, 2013). Because of IBD, the differentiation among populations is predicted to increase with increasing geographic distance due to limited gene exchange and different selection forces (Sexton, Hangartner, & Hoffmann, 2014; Wright, 1943). Based on IBE prediction, populations exchange genes more frequently with populations from the similar conditions than with those from different environments, and experience the same selection pressures and evolve concurrently by local adaptation (Sexton et al., 2014; Wang, 2013). IBD and IBE are not two mutually exclusive mechanisms, as the geographic distance and environmental distance are often correlated (Shafer & Wolf, 2013; Wang, 2013) and play an important role both in native (e.g., the review by Sexton et al., 2014) and introduced species (Alexander, Poll, Dietz, & Edwards, 2009; Cao, Wei, Hoffmann, Wen, & Chen, 2016; Henry et al., 2009; Wu, Yu, Li, & Xu, 2016).

Another factor that contributes to the spatial distribution of genetic diversity is anthropogenic activities. Human‐made habitats can provide windows of opportunity for the establishment of introduced species that may not find other suitable or available habitats in the introduced range (the disturbance hypothesis, Hierro et al., 2005; Hobbs & Huenneke, 1992). Human activities also provide dispersal corridors for alien organisms at either local or global scale (Bradley, Blumenthal, Wilcove, & Ziska, 2010; Bradley, Wilcove, & Oppenheimer, 2010; Moore, 2004). Increased globalization and worldwide trade can in fact facilitate gene exchange within the introduced range, or even between the native and introduced ranges (e.g., by multiple introductions) and can thus mitigate the effects of IBD and IBE. Similar to IBE, gene exchange/dispersal rates are higher among populations occurring in habitats shaped by similar levels of human impact because of isolation‐by‐human activities (IBH). To our knowledge, few studies have investigated the role of anthropogenic factors in gene flow patterns and the relative influence of geographic and climatic variations and human activities on the spatial genetic patterns underpinning the expansion and distribution of invasive species (Wang, Glor, & Losos, 2013).

In this study, we used the common reed, *Phragmites australis* (Cav.) Trin. ex. Steud., to identify the relative contributions of geographic, environmental, and anthropogenic factors to the formation of the patterns of spatial genetic variation. *Phragmites australis* is a wetland perennial grass with a worldwide distribution (Clevering & Lissner, 1999; Guo, Lambertini, Li, Meyerson, & Brix, 2013). In the last several decades, *P. australis* dramatically expanded its distribution in North America (Chambers, Meyerson, & Saltonstall, 1999; Meyerson, Viola, & Brown, 2010; Saltonstall, 2002). The genetic work of Saltonstall (2002) showed that the expansion is due to the introduction of a European lineage, haplotype M, which first appeared in the North America herbarium record ~150 years ago, and has outcompeted the native *P. australis* lineage throughout the continent. Lambertini, Mendelssohn et al. (2012) and Lambertini, Sorrell, Riis, Olesen, and Brix (2012) documented genetic differentiation between the introduced North American and native European populations, and with common garden experiments, Guo et al. (2014) revealed that post‐introduction evolution occurred with the invasion, and Pyšek et al. (2018) identified the differences in genome size as a key trait associated with invasiveness of the common reed populations. In addition, many studies have shown that the establishment of the introduced *P. australis* European lineage in North America is associated with physically disturbed habitats (Bart, Burdick, Chambers, & Hartman, 2006; Meyerson, Saltonstall, & Chambers, 2009; Saltonstall, 2002) and human activities, in particular the occurrence of highways (Jodoin et al., 2008; Lelong, Lavoie, Jodoin, & Belzile, 2007). Guo et al. (2013) detected that human activities have a stronger effect than climate on the distribution of the invasive lineage in North America. Geographic isolation due to the Atlantic Ocean separating the native and introduced ranges could cause strong IBD, and different climates (Guo et al., 2013) could also produce significant IBE between the two ranges. Meanwhile, the strong signal of human activities on *P. australis* distribution and dispersal can mediate these two patterns via IBH. *Phragmites australis* thus provides an ideal model to study the evolutionary mechanisms involved in plant invasions (Eller et al., 2017; Meyerson, Cronin, & Pyšek, 2016).

Using a representative set of samples from the North American and European ranges, we investigated the spatial genetic structure variation patterns within and between the introduced North American (haplotype M, hereafter “NA invasive”) and native European (haplotype M, hereafter “EU native”) populations using microsatellite (SSR) and AFLP markers. Specifically, we asked the following questions: (1) How are populations genetically structured in the native and introduced ranges and do their patterns differ? and (2) How are spatial genetic patterns influenced by geographical distance, climatic variation, and different intensities of human activities within and between ranges?

## 2 MATERIALS AND METHODS

### 2.1 Plant material

**Figure 1:**
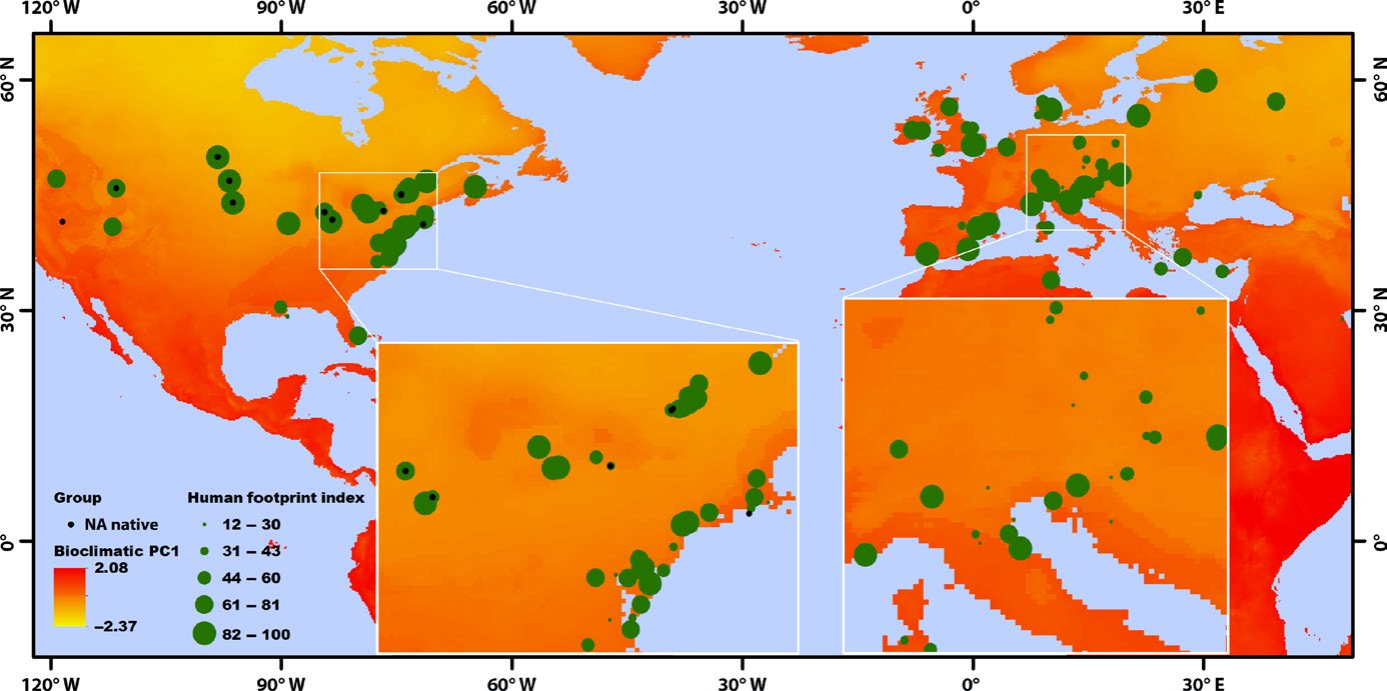
Map of sampling locations of *Phragmites australis*. Different sizes of the green circles are proportional to the human footprint index of the sampling location. Green dots on each continent represent North American (NA invasive, *n* = 92) and European (EU native, *n* = 74) samples, respectively, and black dots are the native North American samples (NA native, *n* = 20). The inset maps highlight the areas of the East Coast of North America (left) and Middle and South Europe (right). The background map of bioclimatic PC1, primarily a temperature‐dominated variable, was derived from Kriticos et al. (2014)

Plant samples used in this study were collected from wild populations in Europe and North America, thereby covering the current distribution range of the species on these continents (Figure 1). All samples were obtained from apical leaves (second leaf or third leaf from the top) and stored separately in bags with silica gel. About 0.5 square centimeters of dry leaf tissue was ground in a mortar with liquid nitrogen, and DNA was extracted with the E.Z.N.A.^®^ SP Plant DNA Kit (Omega Bio‐Tek, USA) according to the manufacturer's instructions for dried specimens.

### 2.2 Cp‐DNA sequences and georeferencing

Two chloroplast intergenic spacer, *trn*T‐*trn*L (Taberlet, Gielly, Pautou, & Bouvet, 1991) and *rbc*L‐*psa*I (Saltonstall, 2001), were amplified in a Peltier Thermal Cycler (PTC‐200 DNA Engine Cycler; MJ Research, St. Bruno, QC, Canada) and sequenced in an ABI sequencer (Applied Biosystems, Foster City, CA, USA), following Saltonstall (2002). Sequences were aligned manually with BioEdit v. 7.1.3.0 (Hall, 1999). After comparing our sequences with the sequences of *P. australis* deposited in GenBank, we found 166 samples belonging to haplotype M (NCBI accession numbers AY016335 for the *trn*T‐*trn*L region and AY016327 for the *rbc*L‐*psa*I region) of which 92 are NA invasive and 74 are EU native, and 20 samples are native North American haplotypes (haplotypes A, AB, AC, E, H) (NA native) (Saltonstall, 2002, 2016). For consistency, we removed the EU samples with sequences different from those of the invasive population in North America, that is, from haplotype M. In total, we included 186 samples in the study. (Detailed information about coordinate and haplotype of each sample is shown in Table S1.) The samples of NA native lineages were used as an outgroup to evaluate the extent of differentiation between EU native and NA invasive and rule out hybridization as a factor that could have a strong spatial, as well as evolutionary, impact on the genetic structure in North America. Although hybridization between NA native and NA invasive occurs, very few hybrids have been detected in wild populations so far (e.g., Meyerson, Lambertini, McCormick, & Whigham, 2012; Meyerson et al., 2010; Saltonstall, Castillo, & Blossey, 2014; Wu, Murray, & Heffernan, 2015).

### 2.3 Nuclear markers

We amplified two AFLP (E‐ACT + M‐CTT, E‐CAG + M‐ATG) and six microsatellites markers (*paGT* 4, *paGT* 8, *paGT* 9, *paGT* 13, Phra 93, and Phra 125) already used in previous studies of *P. australis* variation (Lambertini et al., 2006; Saltonstall, 2003; Yu, Zhang, Ren, & Sun, 2013). These specific primers were chosen for their variability and consistent amplification in a subset of samples representing our sample set. Neutral markers diverge as a result of genetic drift which is indirectly a result of a reproductive barrier due to either ecological or geographic isolation (i.e., IBE, IBH, and/or IBD) (Kirk, Paul, Straka, & Freeland, 2011).

The AFLP protocol was adapted from Vos et al. (1995). Restriction digestion and adapter ligation were performed simultaneously on 100 ng of genomic DNA, with 2.5 units of restriction enzymes EcoRI and of MseI for each, and 1 unit of T4 DNA ligase to ligate 5 pmol EcoRI and 50 pmol MseI double‐stranded nucleotide adapters. Digestion and ligation were performed in a Peltier Thermal Cycler PTC‐200, programmed for 4 hr at 37°C followed by a 0.1°C/s decrease to 16°C in 2 hr, then 70°C for 10 min. The ligated DNA was used for preamplification after four‐time dilution. Preamplification and selective amplification were performed in a Peltier Thermal Cycler PTC‐200. Preamplification was programmed for 20 cycles, each with 30 s DNA denaturation at 94°C, 1 min primer annealing at 56°C, and 1 min DNA extension at 72°C. Selective amplification was programmed at 94°C for 30 s, 65°C for 30 s decreased by 0.7°C/cycle for the subsequent 12 cycles, and 72°C for 1 min, followed by 23 cycles at 94°C for 30 s, 56°C for 30 s, and 72°C for 1 min.

The microsatellite protocols were adapted from Lambertini, Mendelssohn et al. (2012) and Yu et al. (2013). Twenty ng of DNA was added to 18 μl mastermix consisting of 10 μl 2xMasermix (VWR International, Arlington Heights, IL, USA), 10 pmol forward and reverse primers, and sterile water to reach the final volume of 20 μl. Amplification was run in a Peltier Thermal Cycler PTC‐200 under the following conditions: 94°C for 3 min, 40 cycles of 94°C for 30 s, annealing temperature for 40 s, 72°C for 40 s, followed by 72°C for 7 min. Annealing temperatures were as follows: 54°C for *paGT* 4; 50°C for primers *paGT* 8, *paGT* 9, *paGT* 13, 56°C for Phra 93, and 60°C for Phra 125.

The PCR products were run in an ABI 3130XL Genetic Analyzer using GeneScan LIZ 500 as the internal size standard (Applied Biosystems). The AFLP E‐primers and the microsatellites primers *paGT* 4 and *paGT* 9 were Fam‐labeled, *paGT* 8, *paGT* 13, and Phra 93 were Pet‐labeled, and Phra125 was Vic‐labeled.

To estimate the error rates and test the reproducibility of the data, we randomly included three to six control samples in every plate which were amplified every time with the other samples in the plate.

### 2.4 Scoring of genetic data

Genotyping of the AFLP markers was performed in three steps. The aligned peaks were analyzed semiautomatically with GeneMarker v. 2.6.3 (SoftGenetics, State College, PA, USA), with the criteria of peak smooth, peak saturation, baseline subtraction, Local Southern sizing method, in the size range of 50–500 base pairs (bp). The fragments with peak height lower than 50 relative fluorescence units (rfu) were not scored due to the possibility of instrument error (Arrigo, Tuszynski, Ehrich, Gerdes, & Alvarez, 2009; Herrmann et al., 2010). The un‐normalized peak height data were then imported into *RawGeno* v. 2.0.1 (Arrigo et al., 2009), an R package (R Core Team, 2016) for binning and scoring AFLP fragments. The maximum bin size was set to 2 bp, the minimum bin size to 1.5 bp, the scoring range was set from 50 to 500 bp, and the minimum peak height threshold was 100 rfu to eliminate low‐intensity peaks. The raw peak heights and loci size tables from *RawGeno* were combined into one table to produce the input matrix of the marker selection algorithm *scanAFLP* v. 1.3 (Herrmann et al., 2010). The genotyping analysis in *scanAFLP* estimated an error rate of 4.47% for primer pair E‐ACT + M‐CTT and of 1.75% for primer pair E‐CAG + M‐ATG. Both errors are within the typical error range of AFLP data (Bonin et al., 2004; Herrmann et al., 2010; Pompanon, Bonin, Bellemain, & Taberlet, 2005).

The allele sizes of the microsatellite loci were aligned automatically with GeneMarker v. 2.6.3 (SoftGenetics), using GeneScan 500 size standard as a size reference. The obtained alignment was checked manually with Geneious R6 v. 6.0.6 (Biomatters Ltd., Auckland, New Zealand). As *P. australis* is a polyploid species (2n ranges from 4× to 12×) (Clevering & Lissner, 1999), and each locus can have a variable number of alleles, it is difficult to resolve allele dosage. We therefore scored the presence/absence of the microsatellite alleles in a binary matrix (a matrix of ones and zeros, treating each allele as a locus) (Lambertini, Mendelssohn et al., 2012). There were two alleles of two samples that were uncertain so we conservatively scored both of them as absent (0 s).

The resulting two presence/absence matrices (one for AFLPs and one for microsatellites) consisted of 244 polymorphic AFLP loci from the two primer pairs and 50 binary loci from the six microsatellites markers. We analyzed the genetic structure resulting from the two datasets both separately and by pooling the data together. As the outputs of the three analyses were similar, we combined the two molecular matrices in one single matrix of 294 markers to increase the resolution of our dataset following Kettenring and Mock (2012). The binary matrix was handled with R script AFLPdat (Ehrich, 2006) to produce a compatible file format for the subsequent analyses.

### 2.5 Climatic environmental data

The climate data for each genotype were extracted from the extended suite of the WorldClim data (Hijmans, Cameron, Parra, Jones, & Jarvis, 2005; Kriticos, Jarošík, & Ota, 2014; Kriticos et al., 2012), which includes 40 bioclimatic variables. Besides the core set of temperature and precipitation (Bio 1–Bio 19), Kriticos et al. (2012) further added 16 variables of solar radiation and soil moisture (Bio 20–Bio 35). Bio 36–Bio 40 represent the first five principal components (PCs) of the 35 bioclimatic variables (Kriticos et al., 2014). These five PCs capture more than 90% of the variance of the full dataset. Herein, we used these five PCs to obtain the climate at the original collection site for each sample (Figure S1).

### 2.6 Human effect data

Instead of using many individual variables that are standard proxies for the effects of human activities, we used a single comprehensive index, the human footprint index, as the effect of human activities on nature. The index is compiled from several human activity layers such as human population density, land use, and human access, and then normalized by biome (Sanderson et al., 2002). The index ranges from 0 (natural areas) to 100% (completely transformed areas). The detailed information on the human footprint index can be found in Ref. (Guo et al., 2013; Sanderson et al., 2002). The human footprint index for each sample was derived from the Global Human Footprint Dataset (v. 2, 1995–2004) (Wildlife Conservation Society—WCS, and Center for International Earth Science Information Network—CIESIN—Columbia University, 2005) (Figure 1). Both bioclimatic and human footprint index layers used had a resolution of 30 arc‐second grid cells.

### 2.7 Statistical analysis

The genetic data were used to (1) infer population genetic structure based on AFLP and SSR markers and (2) correlate genetic data with geographic (IBD), climatic (IBE), and human impact (IBH) data and assess the contribution of IBD, IBE, and IBH to gene flow within and between ranges by path analysis and hierarchical partitioning.

#### 2.7.1 Population genetic structure

The multivariate discriminant analysis of principal components (DAPC, Jombart, Devillard, & Balloux, 2010) seeks linear combinations between data (binary loci in our study), which maximize differences between groups while minimize variation within groups. It first performs a principal component analysis (PCA) of the genetic binary dataset and then runs the discriminant analysis (DA) with the PCA components as the input variables. In addition, the analysis can derive probabilities for each individual of membership in each of the different resulting clusters based on the retained discriminant functions. The DAPC was performed using the R package *adegenet* (Jombart, 2008) with the binary matrix. By inferring a maximum cluster number (*K*), the package runs the *K*‐means clustering algorithm sequentially and identifies an optimal number of genetic clusters via the Bayesian information criterion (BIC) within the inferred *K* range. We ran DAPC twice, first with all three populations (EU native, NA invasive, and NA native), and then without NA native, to detect the structure of the native and introduced populations of the invasive European lineage. The maximum *K* was set to 40 for both runs.

In addition, we also inferred population structure and population assignment simultaneously with a Bayesian Markov Chain Monte Carlo (MCMC) clustering approach implemented in STRUCTURE v. 2.3.4 (Falush, Stephens, & Pritchard, 2007; Pritchard, Stephens, & Donnelly, 2000). Like for DAPC, we first run the analysis with all three populations with *K* set from one to eight, and subsequently without the NA native group with *K* from one to six. For each *K* value, we ran 10 replicates with 300,000 burn‐in iterations and 1,000,000 MCMC iterations. For both STRUCTURE analyses, we chose the admixture model and correlated allelic frequencies, and no prior information on individual's origin. We followed the method of Evanno, Regnaut, and Goudet (2005) and used the ad hoc statistic ∆*K* as the criterion to identify the most likely number of clusters (*K*), that is, when *∆K* is highest. This test was run with STRUCTURE HARVESTER (Earl & Vonholdt, 2012). CLUMPAK (Kopelman, Mayzel, Jakobsson, Rosenberg, & Mayrose, 2015) was then used to merge and visualize the results.

We finally compared genetic diversity (calculated as number of alleles, Shannon information index, and gene diversity) between NA invasive and EU native and quantified the number of shared and distinct alleles between the two ranges (with GenAIEx, Peakall & Smouse, 2006, 2012) in order to evaluate the relevance of founder effect and/or bottlenecks.

#### 2.7.2 Gene flow patterns within and between ranges

##### Mantel tests

We tested for IBD within and between the NA and EU populations with a Mantel test, which tests the correlation between pairwise Euclidean genetic distance and Euclidean geographic distance (km) among the individuals of each population with GenAIEx 6.5 (Peakall & Smouse, 2006, 2012). Statistical support was obtained with 1,000 permutations.

We then tested the correlation between Euclidean genetic distances and pairwise Euclidean bioclimatic distance (to assess IBE), and between Euclidean genetic distances and pairwise Euclidean distances in human footprint index (to assess isolation by human effects, IBH), respectively.

##### Path analysis

Path analyses were used to quantify the relative contributions of geographical distance (IBD), bioclimatic environmental dissimilarity (IBE), and human influence distance (IBH) to the genetic structure within each range and between ranges (NA invasive vs. EU native) (Wang et al., 2013). As a statistical framework for evaluating complex relationships between multiple variables, path analysis uses a series of regression and model‐fitting analyses to calculate the correspondence of a priori defined relationships among variables simultaneously, and can provide the standardized coefficients indicating the magnitudes of the relationships between variables (Grace, 2006; Wang et al., 2013).

Path analyses were performed using *lavaan* package (Rosseel, 2012) in R. Model fit was evaluated by χ^2^ test, the root mean squared error of approximation (RMSEA), standardized root mean square residual (SRMR), and the comparative fit index (CFI). Nonstatistical significance (*p* > .05) of the χ^2^ test indicates that the model significantly simulated the data. Values of RMSEA smaller than 0.05 indicate a very good model fit (Finch & Frenc, 2015). The 90% confidence interval (CI) of RMSEA assesses the precision of the RMSEA estimate, and the lower boundary (left side) of the CI shall be, or be very close to, zero and the upper boundary (right side) be <0.08 for a close fit (Schermelleh‐Engel, Moosbrugger, & Müller, 2003). SRMR ranges from zero to one, and values lower than 0.8 are considered well‐fitting models (Hu & Bentler, 1999). A CFI value higher than 0.90 indicates a very good model fit (Finch & Frenc, 2015). If none of the significance criteria was reached, we ran a saturated model first. A saturated model, that is, one in which all variables are correlated with each other, has the best possible fit as it perfectly reproduces variances, covariances, and means. The saturated model was used as a standard for comparison with other estimated models via AIC values. Modification indices (MI) were used in our study to detect potential paths that can be added to the model to improve the goodness of the model fit. We set the bootstrap value to 1,000 to calculate the CIs for parameter coefficients in each model. To compare the relative contribution of each distance and improve normality, the four distances (genetic, geographic, environmental, and human activities) were log‐transformed prior to analysis.

##### Hierarchical partitioning

We also analyzed the independent effect of geography, bioclimatic environment, and human influence on genetic distances via independent effect analysis (hierarchical partitioning) for between and within the European and North American ranges. As a method of multiple regression, independent effect analysis estimates the average contribution of each explanatory variable to the variance of the response variable by testing all possible models that represent a subset of the explanatory variables (Chu et al., 2016; Murray & Conner, 2009), and is appropriate and effective to pinpoint the most likely causal factors while alleviating multicollinearity problems. Independent effect analysis was carried out in *hier.part* package (Olea, Mateo‐Tomas, & de Frutos, 2010).

## 3 RESULTS

### 3.1 Genetic structure

**Figure 2:**
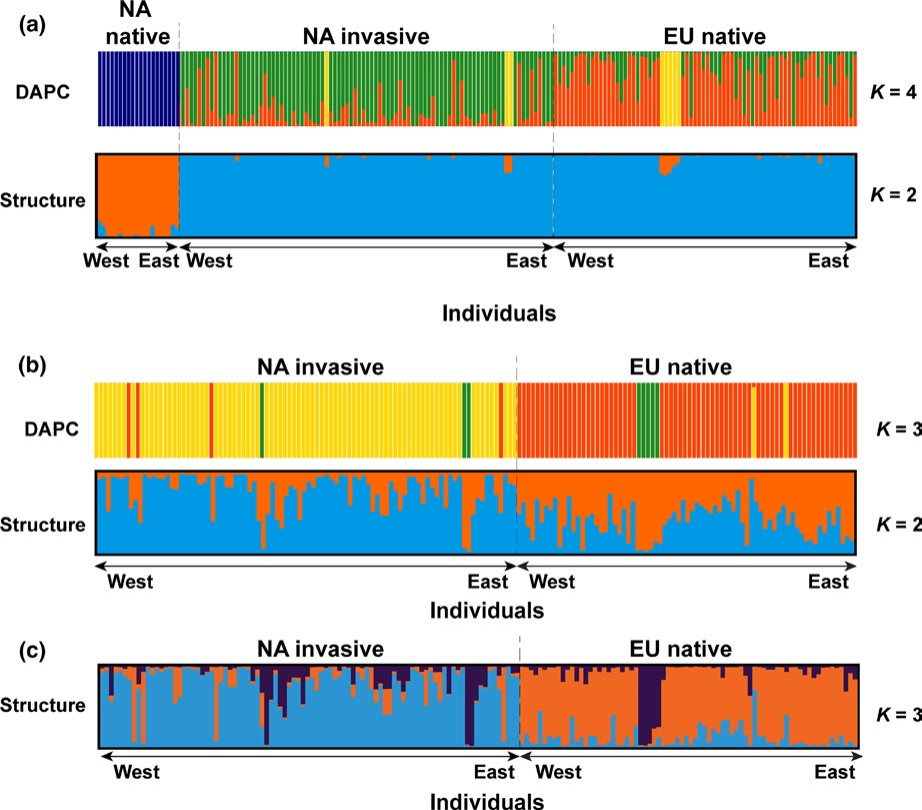
(a) Three‐group DAPC and STRUCTURE analyses of the molecular data for individuals of *Phragmites australis*; (b) two‐group (without NA native) DAPC and STRUCTURE analyses of the molecular data for individuals of *P. australis*; (c) substructure (STRUCTURE analyses) of the two‐group *P. australis*. Individuals are sorted from west to east within each population. Different colors indicate different ancestral groups. Phylogeographic groups are separated by dashed lines. Inferences of the best number of ancestral groups are shown in Figure S2

STRUCTURE *∆K* was highest for *K *=* *2 (Figure 2a; Figure S2c), indicating the presence of two ancestral groups for the three lineages, that is, one NA native and one for haplotype M including both NA invasive and EU native (Figure 2a, STRUCTURE, *K *=* *2). Contrary to STRUCTURE, the lowest BIC of DAPC analyses indicated four clusters in the whole dataset (Figures S2a and S3a). In addition to the original three groups of NA native, NA invasive, and EU native, DAPC resolved a fourth common ancestral group for eight individuals collected in Denmark and the East Coast of North America (Figure 2a). After removing the native North American samples, DAPC constantly found three ancestral groups for the haplotype M samples (Figure 2b; Figures S2b and S3b), while STRUCTURE revealed a structure (*K *=* *2) corresponding to NA invasive and EU native and a substructure (*K *=* *3) in agreement with DAPC (Figure 2c; Figure S2d). Both STRUCTURE and DAPC analyses certified that the native North American samples had a distinct origin compared to haplotype M samples, and EU native and NA invasive had a common ancestor. Both analyses identified also a new third group within haplotype M consisting of the eight samples from Denmark and the East Coast of North America (Figure 2). Given the small number of samples and the new discovery of this group, these eight genotypes were removed for further analysis to decrease the bias in the isolation patterns investigated in our study. After removing the divergent genotypes, NA invasive shared 68% for SSR alleles and 86% of AFLP alleles with EU native and had lower, although comparable, genetic diversity to EU native (Table 1).

**Table 1:** Descriptive parameters of genetic variations per group

	*N*	Ne	*I*	He	uHe
NA invasive	89	1.213 (0.017)	0.227 (0.013)	0.138 (0.009)	0.139 (0.009)
EU native	69	1.227 (0.018)	0.241 (0.013)	0.146 (0.009)	0.147 (0.010)

*N*, sample size; Ne, No. of effective alleles; *I*, Shannon's information index; He, expected heterozygosity, uHe, unbiased expected heterozygosity.

Data are mean (*SE*).

### 3.2 Gene flow patterns within and between ranges

**Figure 3:**
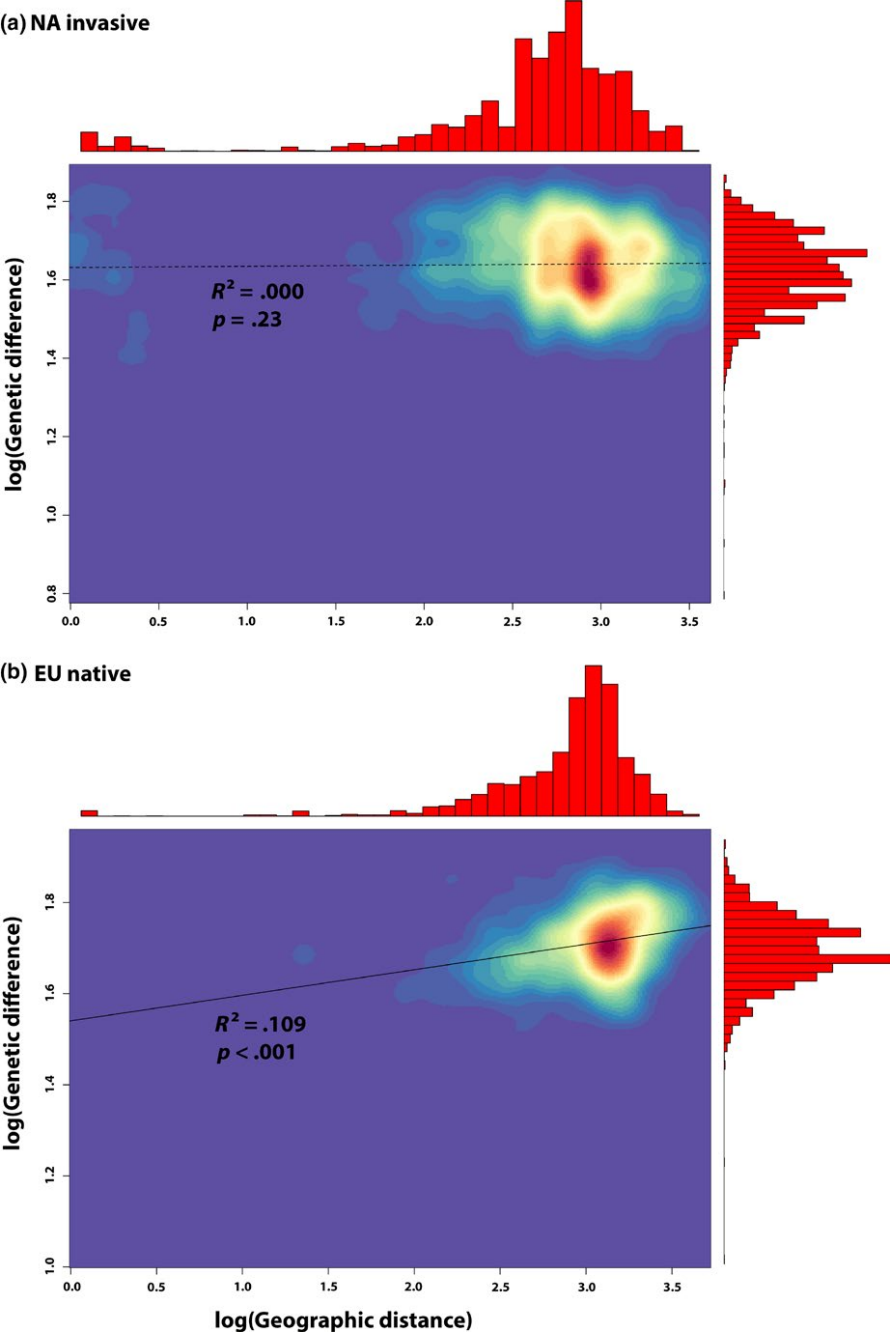
Density plot of the relationship between geographic distance and genetic distance (IBD)

**Figure 4:**
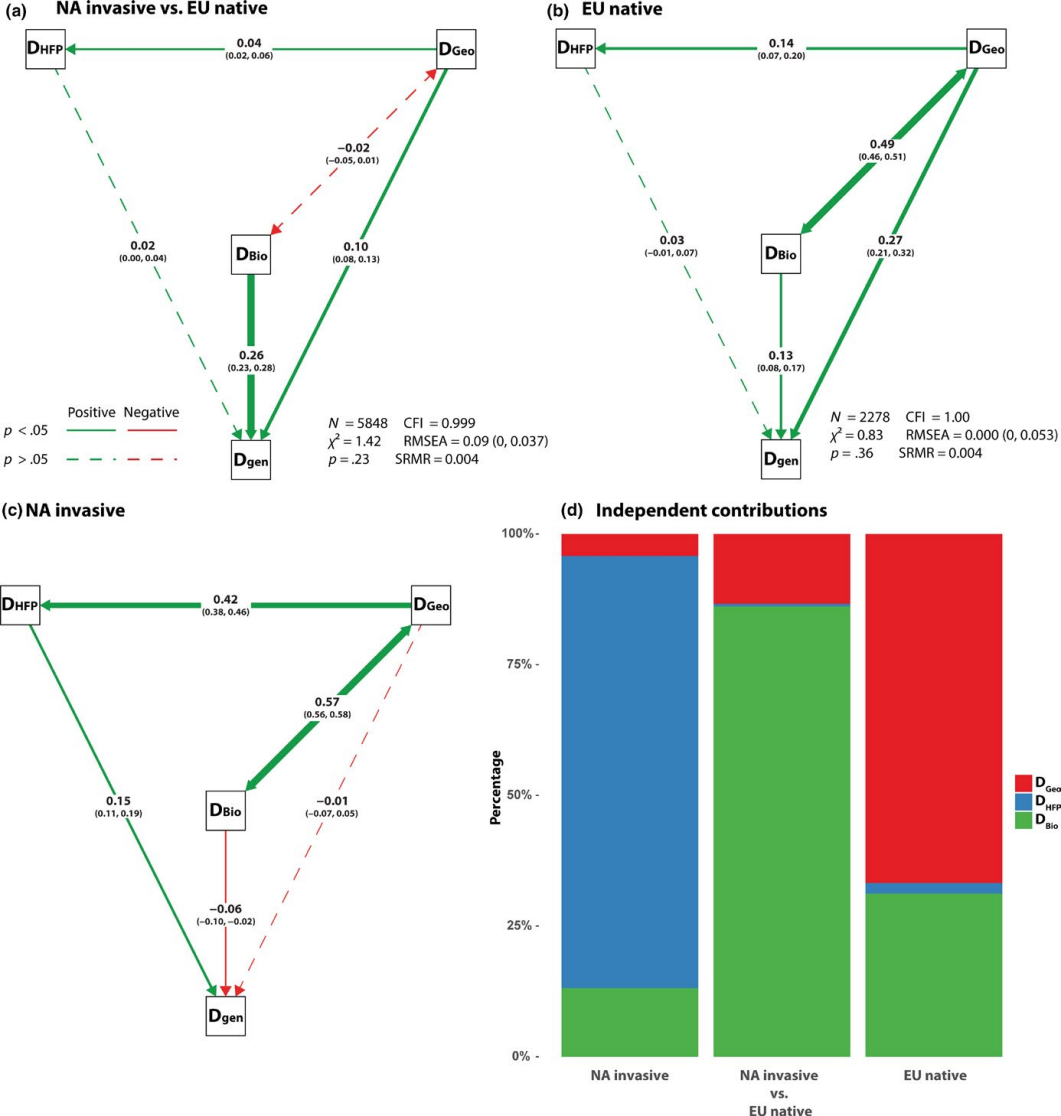
Path analyses to determine the relative contributions of geography, climate, and human effects to differentiation of NA invasive from EU native (a), within EU native (b), and within NA invasive (c). *D*_Bio_, bioclimatic distance; *D*_Geo_, geographic distance; *D*_gen_, genetic distance; and *D*_HFP_, human footprint index distance. The one‐way arrow in the model indicates causal relationships, and the two‐way arrow indicates correlation. The solid red arrow represents negative path (*p *<* *.05), solid green arrows represent positive paths (*p *<* *.05), and dashed arrows represent nonsignificant paths (*p *>* *.05). The numbers on the arrows are the standardized path coefficients, and the numbers in brackets are the 97.5% CIs of the coefficients. The width of the arrows is proportional to the value of the path coefficient. *N*, number of samples; CFI, comparative fit index; RMSEA, root mean square error of approximation (90% CI); SRMA, standardized root mean square residual. For (c) NA invasive, the model showed did not reach any evaluation criteria; however, the model did not differ from the saturated model of the data. (d) Results of the randomization tests of the independent contributions of separate predictor variables (hierarchical partitioning) explaining variation in genetic distances between ranges (NA invasive vs. EU native), within Europe (EU native), and within North America (NA invasive)

The IBD revealed a significant positive relationship between geographic and genetic distances (*p *<* *.001) for the EU native but not for the NA invasive (*p *=* *.23) (Figure 3). These results were corroborated also by path analysis and hierarchical partitioning analysis (Figure 4b–d). Path analysis found significantly positive IBD and IBE for EU native (Figure 4b), and a negative IBE and a positive IBH for NA invasive (Figure 4c). When we considered NA invasive versus EU native, the path analysis showed positively significant effects of both IBD and IBE, and the coefficient of IBE was 2.6 times higher than that of IBD (Figure 4a). Hierarchical partitioning analysis also showed that bioclimatic distances (*D*_Bio_) contributed most (86% of the total variation) to the genetic distances between native and introduced ranges, while within EU native and NA invasive populations, geographic distances (*D*_Geo_) and human footprint distances (*D*_HFP_) were the primary contributors to the genetic distance within population, respectively (Figure 4d).

## 4 DISCUSSION

### 4.1 Genetic structure and isolation between ranges

Novel environmental conditions in the introduced range may act as strong selection forces on some introduced species and lead to rapid evolution within the time frame of centuries or even decades (Colautti & Lau, 2015). The lack of frequent gene flow between the native and introduced ranges of the introduced population due to isolation also tends to cause genetic differentiation between ranges, and may eventually lead to allopatric speciation (Bouton, 2000; Endler, 1977; Taylor & McPhail, 1999). Using samples of the *P. australis* haplotype M lineage from its introduced and native ranges, we found that the invasive North American and native European populations genetically diverged, although they still share the majority of their alleles. This result was confirmed by all analyses performed, that is, DAPC and STRUCTURE, with both IBD and IBE (represented by climate in our study) playing a role. The loss of genetic diversity due to founder effect is comparable to that calculated by Dlugosch and Parker (2008) in 20 invasive plant species. As shown by these authors, the diversity loss changed over time, depending on gene flow opportunities and the occurrence of multiple introductions, and tended to decrease over a timescale comparable to the introduction of *P. australis* in North America. The founder effect did not have a direct effect on quantitative trait diversity and the ability of evolving adaptive potential, at least for the modest genetic diversity losses as measured by Dlugosch and Parker (2008).

Sexton et al. (2014) showed that non‐native plant populations predominantly exhibit IBD and IBE patterns of gene flow due to limited dispersal ability following introduction to the new range, and local adaptation. We found significant effects of IBD on populations of the European lineage between ranges, indicating that the genetic difference between the North American and European populations resulted in part from the long‐distance transport between the two continents and from the natural barrier (the Atlantic Ocean) between them. However, we found that IBE, in our case represented by climate, was a more important mechanism than IBD in terms of contributing to the differentiation between ranges. This indicates that nonanalogous climates in the two ranges contributed more than allopatry and founder effect to the genetic differentiation of the populations in the two ranges. This is in agreement with Guo et al. (2013) who found that the climatic niche had shifted between the native and introduced populations of the invasive European lineage. Niche shifts can be a potent selection force and cause rapid evolution in the new range. Guo et al. (2014) found that photosynthesis‐ and growth‐related traits of the invasive North American populations became different from the ancestral European populations, possibly as a response to the new niche (Guo et al., 2013). We cannot, however, discount the possibility that the differences that we observed could also be due to introductions of different populations from Europe originating from a variety of environmental conditions. We did not find any significant relationship between the differences in human activity between the two ranges and genetic distance, which suggests that the effects of human influence on native and introduced haplotype M populations analyzed in our study did not fundamentally differ. The similarity between genotypes on the East Coast of North America and Denmark samples removed from the data analysis could erroneously be taken as an evidence of multiple introductions from Europe to North America. Because the source population of these samples is unknown, their origin must be investigated further before any conclusions can be drawn.

### 4.2 Mechanisms acting within each range

The European lineage of *P. australis* showed different genetic structure patterns within its native and introduced ranges. In the native European range, gene structure of *P. australis* populations reflected a positive IBD and IBE, a classic isolation pattern shaped by dispersal distance and environmental variations common to most wild species (Sexton et al., 2014; Shafer & Wolf, 2013). In contrast, we did not detect a significant effect of IBD in the introduced range in North America, indicating that there is no significant geographic barrier to the dispersal of the introduced genotypes. Nevertheless, the introduced populations showed a weak, yet significant negative IBE and a strong positive IBH. The negative IBE implies that gene flow among dissimilar environments, that is, areas with contrasting climates, was greater than would be expected due to random gene flow. This gene flow scenario has previously been defined as a counter‐gradient gene flow and can be caused by directional gene flow, usually due to human infrastructure, which can interconnect populations that would otherwise be isolated because of distance or ecology (Kirkpatrick & Barton, 1997; Sexton et al., 2014). Considering the strong positive IBH in the introduced populations, the counter‐gradient gene flow could either be the result of human‐mediated dispersal along coastlines, roads, railroads, and navigable rivers, which are an important part of the human footprint index calculated in our study (Sanderson et al., 2002), or result from unintentional preselection in Europe, as the European populations from areas with high human activities can have a high chance to be selected and introduced to other ranges. Introduced *P. australis* can reproduce both sexually (Haslam, 1972) and vegetatively and can be dispersed (via either seeds or propagules) over long distances through waterways (Kirk et al., 2011; Meyerson, Pergl, & Pyšek, 2014) and highways (e.g., Bart et al., 2006; Catling & Carbyn, 2006; Jodoin et al., 2008; LeBlanc, De Blois, & Lavoie, 2010; Lelong et al., 2007).

Theory predicts that under counter‐gradient gene flow, the strong directional gene transfer could prevent local adaptation (Sexton et al., 2014; Sultan & Spencer, 2002). This is contrary to the strong IBE that we found between the introduced and native ranges, which suggests that either local adaptation to climate has occurred in the introduced range or the introduced genotypes were preadapted prior to the introduction. The *P. australis* haplotype M lineage was introduced into the East Coast of North America at least 150 years ago, but it started to become widely dispersed in middle and western North America only in the last half century (Kulmatiski, Beard, Meyerson, Gibson, & Mock, 2011; Saltonstall, 2002); in a few states, the invasion was recognized only recently (Guo et al., 2013; Kettenring & Mock, 2012; Melchior & Weaver, 2016). This suggests that local adaptation might have occurred on the East Coast before the introduced population spread westward (Allen et al., 2017; Bhattarai et al., 2017). This is in agreement with conclusions of McCormick, Kettenring, Baron, and Whigham (2010) who suggested that the lag time between the introduction and expansion of invasive *P. australis* in North America could be due to Allee effect, that is, the time needed for the introduced genotypes to build up population density and fitness before spreading invasively across the continent. Several recent studies confirm that the introduced *P. australis* populations heavily rely on sexual reproduction (Belzile, Labbé, LeBlanc, & Lavoie, 2010; Pyšek et al., 2018). By dispersing pollen and/or seeds widely, the likelihood of outcrossing (Kettenring & Whigham, 2009; Kirk et al., 2011; McCormick et al., 2010; Meyerson et al., 2010) increases and the Allee effect is self‐enforced, as well as the adaptive potential of the introduced population.

## 5 CONCLUSIONS

The invasive populations of *P. australis* in North America have evolved from their ancestral populations in Europe. A different climate in the two ranges contributes the most to the genetic differentiation of the two populations, followed by geographic isolation. Within the two ranges, geographic distances, climatic variation, and human impact have shaped the genetic patterns differently. In the native range in Europe, the population structure has been shaped since postglacial colonization (e.g., Ingrouille, 1995), mainly by geographic barriers and climatic variation, whereas in the introduced range in North America, human‐made habitats have prevented IBD from having an effect on structuring the populations, and facilitated counter‐gradient gene flow. Because of the strong IBE between the native and introduced range, despite counter‐gradient gene flow within the introduced range, the evolutionary scenario that better explains the invasion in North America is that of rapid post‐introduction evolution of founding populations, possibly by genetic drift, founder effects, Allee effect and local adaptation to climate and human‐made habitats, and dispersal of adapted genotypes throughout the continent. More in‐depth sampling and research are needed to investigate the roles of preadaptation, multiple introductions, and interactions with the existing communities, factors which can also contribute to the observed spatial patterns.

## CONFLICT OF INTEREST

None declared.

## AUTHOR CONTRIBUTIONS

W.‐Y.G. and C.L. designed the experiments. W.‐Y.G. analyzed the data. W.‐Y.G. and C.L. wrote the manuscript with contributions from all authors.

## DATA ACCESSIBILITY

Microsatellite matrix and AFLP genetic matrix have been archived in the Dryad Digital Repository: https://doi.org/10.5061/dryad.fj46f.
