# Supplementary material for: Living in two worlds: Evolutionary mechanisms act differently in the native and introduced ranges of an invasive plant
Source: Ecol Evol. 2018 Jan 29;8(5):2440–52. doi: 10.1002/ece3.3869 (PMC5838077; doi:10.1002/ece3.3869)
Supplement: Supplementary file 1 [file ECE3-8-2440-s001.docx]

**Supplementary materials**


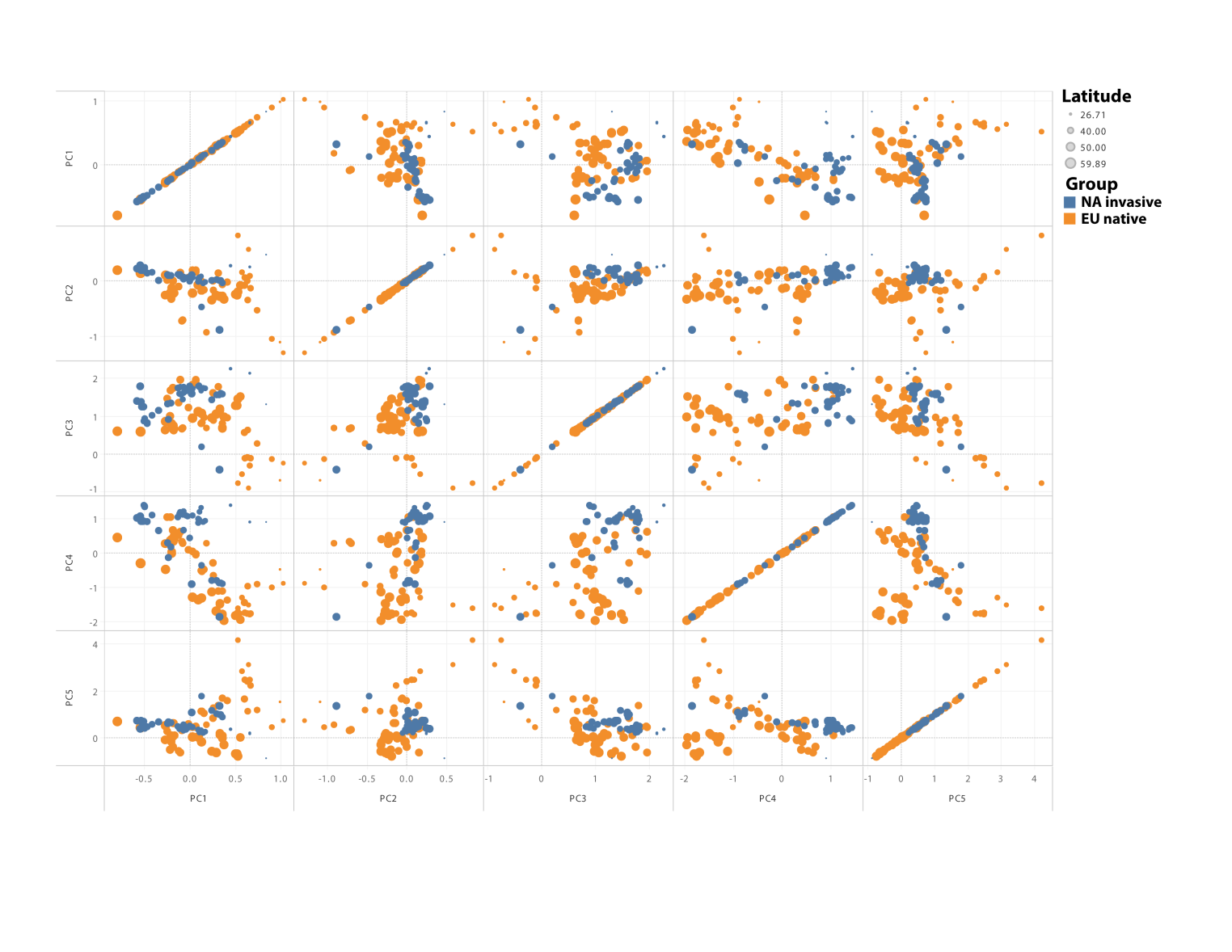


**Fig. S1** Scatter plots of the first five bioclimatic PCs of the introduced North American and native European populations. The sizes of the dots represent the value of latitude.


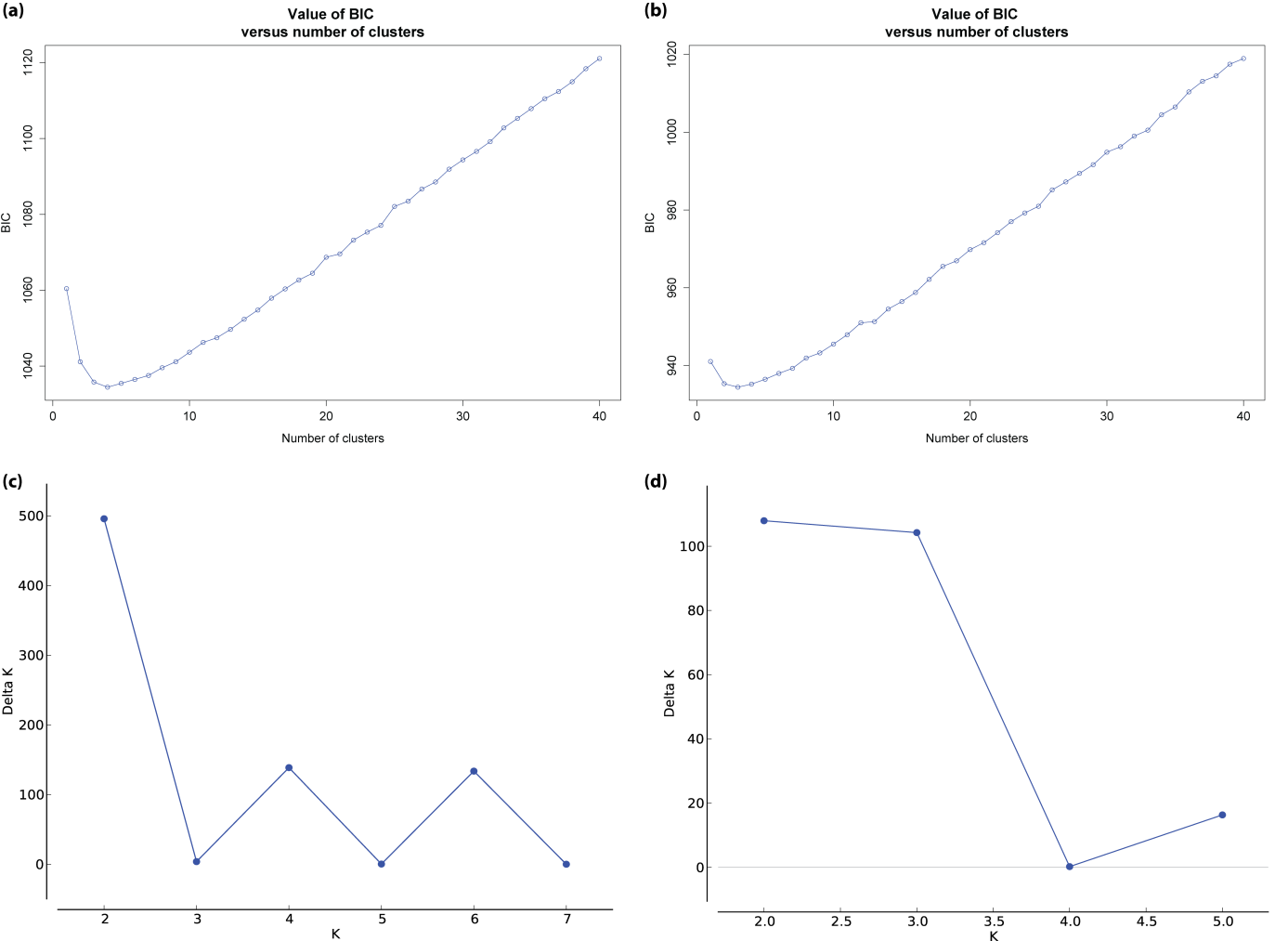


**Fig. S2** Inference of the number of clusters based on the BIC value of the DAPC performed with three groups (EU native, NA invasive, and NA native) (a) and with two groups (EU native and NA invasive) (b), and inference of the *ΔK* values of STRUCTURE analysis of the three groups (c) and the two groups (d).


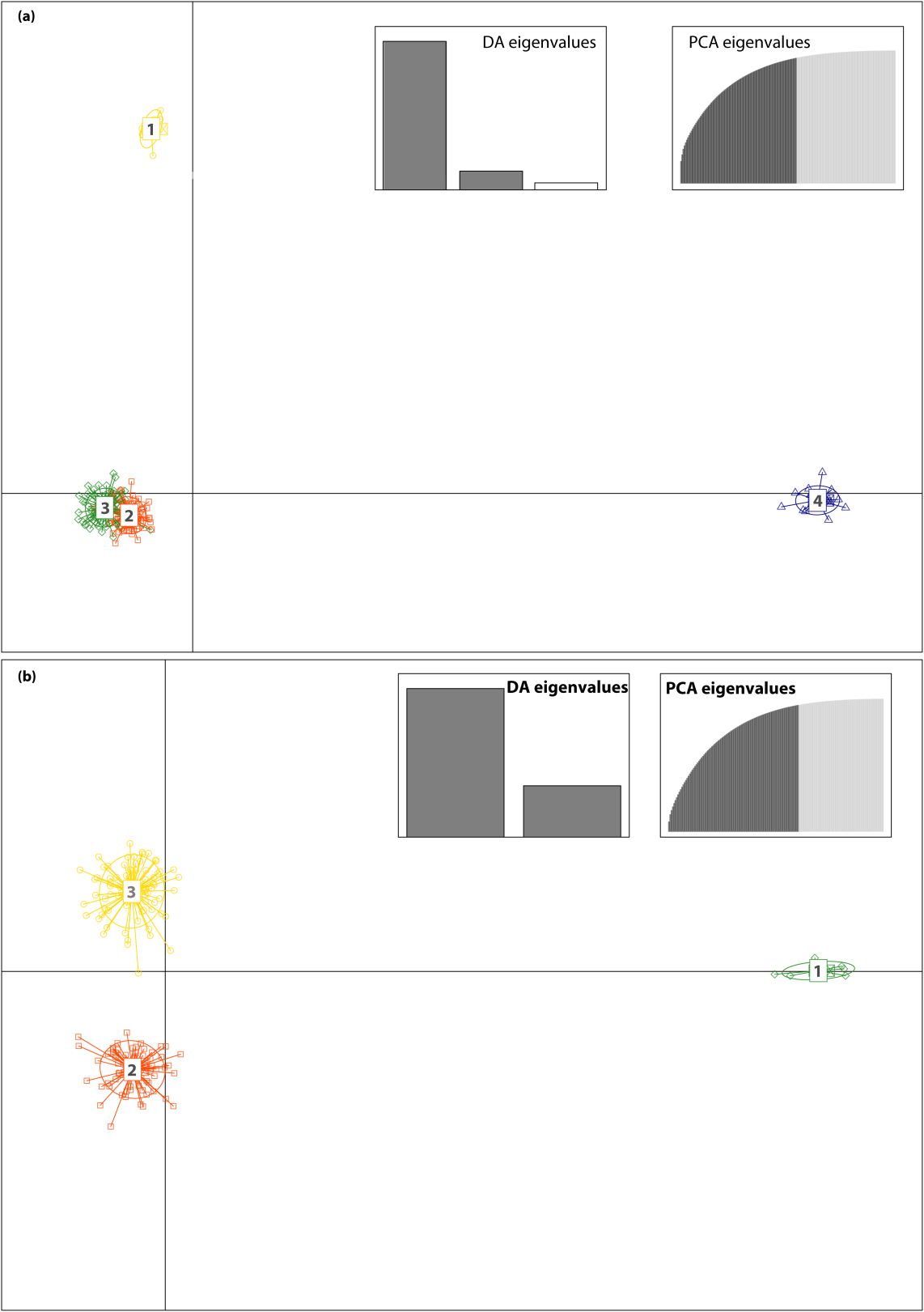


**Fig. S3** Discriminant analysis of principal components (DAPC) ordination of the clusters by the first two axes using molecular data. Dots represent individuals. (a) three-group DAPC analysis, (b) two-group DAPC analysis. Colors of the clusters correspond to the colors of the groups in Fig. 1b and 1c. Cluster 1 includes samples from Denmark and the east coast of North America, cluster 2 includes EU native samples, cluster 3 includes NA invasive samples, and cluster 4 includes NA native samples. The circles are 95 % confidence ellipses.

**Table S1** Sampling used in this study, including individual IDs, group of origin based on haplotype and coordinates. NA invasive, introduced North American *P. australis*; NA native, native North American *P. australis*; EU native, native European *P. australis*.

| **Individuals** | **Group** | **Haplotype** | **Latitude** | **Longitude** |
| --- | --- | --- | --- | --- |
| GC128Luci | NA invasive | M | 26.707 | -80.036 |
| GCH143 | NA invasive | M | 30.404 | -90.157 |
| US99 | NA invasive | M | 36.270 | -77.590 |
| US194 | NA invasive | M | 36.853 | -75.978 |
| US210 | NA invasive | M | 37.208 | -76.774 |
| US186 | NA invasive | M | 37.286 | -75.923 |
| US189 | NA invasive | M | 37.784 | -75.600 |
| US69 | NA invasive | M | 38.576 | -75.263 |
| US115 | NA invasive | M | 38.772 | -76.083 |
| US182 | NA invasive | M | 38.775 | -75.110 |
| US185 | NA invasive | M | 38.794 | -77.288 |
| US203 | NA invasive | M | 39.051 | -74.758 |
| US179 | NA invasive | M | 39.158 | -75.525 |
| US187 | NA invasive | M | 39.297 | -75.177 |
| US192 | NA invasive | M | 39.297 | -75.177 |
| US193 | NA invasive | M | 39.297 | -75.177 |
| US181 | NA invasive | M | 39.457 | -75.662 |
| US180 | NA invasive | M | 39.575 | -75.707 |
| US112 | NA invasive | M | 39.938 | -74.389 |
| US206 | NA invasive | M | 41.222 | -73.051 |
| US61 | NA invasive | M | 41.335 | -89.110 |
| US113 | NA invasive | M | 41.377 | -71.511 |
| US114 | NA invasive | M | 41.563 | -83.654 |
| US128 | NA invasive | M | 41.606 | -70.857 |
| US200 | NA invasive | M | 41.789 | -71.368 |
| US199 | NA invasive | M | 42.491 | -71.277 |
| US190 | NA invasive | M | 42.886 | -78.879 |
| US71 | NA invasive | M | 42.886 | -78.879 |
| US197 | NA invasive | M | 42.904 | -78.693 |
| US191 | NA invasive | M | 43.276 | -77.278 |
| CA129 | NA invasive | M | 43.667 | -79.417 |
| CA131 | NA invasive | M | 45.083 | -74.183 |
| CA153 | NA invasive | M | 45.133 | -74.000 |
| CA155 | NA invasive | M | 45.233 | -73.800 |
| CA151 | NA invasive | M | 45.500 | -73.583 |
| CA152 | NA invasive | M | 45.567 | -73.850 |
| CA154 | NA invasive | M | 46.033 | -73.433 |
| CA132 | NA invasive | M | 46.800 | -71.167 |
| US116 | NA invasive | M | 47.119 | -119.268 |
| GCH127 | NA invasive | M | 29.142 | -89.222 |
| GC124 | NA invasive | M | 29.160 | -89.234 |
| GC128Missi | NA invasive | M | 29.166 | -89.269 |
| GC133 | NA invasive | M | 29.232 | -89.252 |
| ROM2 | NA invasive | M | 29.260 | -89.241 |
| US1005 | NA invasive | M | 38.878 | -76.545 |
| US1006 | NA invasive | M | 38.878 | -76.545 |
| US1004 | NA invasive | M | 38.878 | -76.545 |
| US1003 | NA invasive | M | 38.878 | -76.545 |
| US999 | NA invasive | M | 38.883 | -76.550 |
| US1002 | NA invasive | M | 38.883 | -76.553 |
| US1000 | NA invasive | M | 38.883 | -76.551 |
| US994 | NA invasive | M | 38.883 | -76.552 |
| US1007 | NA invasive | M | 38.883 | -76.548 |
| US995 | NA invasive | M | 38.883 | -76.554 |
| US1001 | NA invasive | M | 38.884 | -76.552 |
| US998 | NA invasive | M | 38.884 | -76.552 |
| US990 | NA invasive | M | 40.769 | -74.064 |
| US986 | NA invasive | M | 40.770 | -74.064 |
| US991 | NA invasive | M | 40.770 | -74.064 |
| US989 | NA invasive | M | 40.770 | -74.064 |
| US985 | NA invasive | M | 40.770 | -74.064 |
| US992 | NA invasive | M | 40.770 | -74.065 |
| US983 | NA invasive | M | 40.770 | -74.065 |
| US984 | NA invasive | M | 40.770 | -74.065 |
| US949 | NA invasive | M | 40.850 | -73.876 |
| US804 | NA invasive | M | 40.871 | -111.939 |
| US947 | NA invasive | M | 41.179 | -71.586 |
| US948 | NA invasive | M | 41.184 | -71.569 |
| US802 | NA invasive | M | 41.216 | -89.323 |
| US803 | NA invasive | M | 41.257 | -89.394 |
| US946 | NA invasive | M | 41.380 | -71.498 |
| US974 | NA invasive | M | 41.380 | -71.493 |
| US975 | NA invasive | M | 41.381 | -71.495 |
| US982 | NA invasive | M | 41.381 | -71.494 |
| US941 | NA invasive | M | 41.657 | -71.419 |
| US942 | NA invasive | M | 41.657 | -71.419 |
| US943 | NA invasive | M | 41.657 | -71.419 |
| GCLK16 | NA invasive | M | 42.757 | -84.382 |
| GCLK11 | NA invasive | M | 42.766 | -84.398 |
| GCLK4 | NA invasive | M | 42.767 | -84.383 |
| GCLK8 | NA invasive | M | 42.767 | -84.393 |
| GCLK5 | NA invasive | M | 42.767 | -84.382 |
| GCLK9 | NA invasive | M | 42.767 | -84.393 |
| GCLK10 | NA invasive | M | 42.767 | -84.393 |
| GCLK17 | NA invasive | M | 42.768 | -84.385 |
| GCLK3 | NA invasive | M | 42.768 | -84.385 |
| GCLK12 | NA invasive | M | 42.768 | -84.402 |
| GCLK13 | NA invasive | M | 42.769 | -84.402 |
| GCLK14 | NA invasive | M | 42.769 | -84.402 |
| CA952 | NA invasive | M | 45.591 | -73.753 |
| US980 | NA invasive | M | 46.074 | -64.783 |
| US993 | NA invasive | M | 46.075 | -64.782 |
| US65 | NA native | E | 41.785 | -83.374 |
| US211 | NA native | E | 44.001 | -96.317 |
| US55 | NA native | E | 46.874 | -96.767 |
| CA204 | NA native | E | 49.967 | -98.301 |
| CA130 | NA native | E | 49.967 | -98.300 |
| US944 | NA native | AC | 41.178 | -71.567 |
| US945 | NA native | AC | 41.180 | -71.566 |
| US940 | NA native | H | 41.517 | -118.488 |
| GCLK2 | NA native | AB | 42.757 | -84.382 |
| GCLK6 | NA native | E | 42.766 | -84.394 |
| GCLK7 | NA native | E | 42.766 | -84.390 |
| GCLK15 | NA native | AB | 42.769 | -84.383 |
| GCLK1 | NA native | AB | 42.769 | -84.383 |
| US938 | NA native | E | 42.959 | -76.742 |
| US939 | NA native | E | 42.959 | -76.742 |
| CA951 | NA native | E | 45.042 | -74.463 |
| US819 | NA native | E | 45.042 | -74.463 |
| US821 | NA native | E | 45.067 | -74.431 |
| US823 | NA native | E | 45.093 | -74.407 |
| US950 | NA native | A | 45.900 | -111.524 |
| KW90 | EU native | M | 28.827 | 47.999 |
| CY671 | EU native | M | 35.037 | 32.426 |
| GR57 | EU native | M | 35.365 | 24.471 |
| ES74 | EU native | M | 40.717 | 0.583 |
| ES95 | EU native | M | 40.998 | -1.507 |
| ES72 | EU native | M | 41.000 | -1.500 |
| RO652 | EU native | M | 45.000 | 29.217 |
| RO650 | EU native | M | 45.000 | 29.217 |
| RO653 | EU native | M | 45.000 | 29.217 |
| RO651 | EU native | M | 45.001 | 29.217 |
| RO655 | EU native | M | 45.001 | 29.217 |
| RO625 | EU native | M | 45.192 | 29.291 |
| RO84 | EU native | M | 45.192 | 29.291 |
| IT207 | EU native | M | 45.683 | 9.767 |
| SL170 | EU native | M | 45.970 | 14.433 |
| SL172 | EU native | M | 46.055 | 14.514 |
| SL79 | EU native | M | 46.055 | 14.514 |
| SL171 | EU native | M | 46.055 | 14.514 |
| HU663 | EU native | M | 47.600 | 17.033 |
| HU77 | EU native | M | 47.646 | 16.743 |
| CZ620 | EU native | M | 48.650 | 14.367 |
| CZ666 | EU native | M | 48.903 | 16.735 |
| CZ676 | EU native | M | 49.602 | 14.714 |
| BE67 | EU native | M | 51.217 | 4.417 |
| NL163 | EU native | M | 51.329 | 4.135 |
| DE640 | EU native | M | 51.433 | 13.617 |
| GB209 | EU native | M | 51.500 | -0.117 |
| PL78 | EU native | M | 51.733 | 18.517 |
| DE639 | EU native | M | 51.817 | 13.817 |
| IR166 | EU native | M | 53.250 | -7.117 |
| IR58 | EU native | M | 53.402 | -6.671 |
| IR164 | EU native | M | 53.433 | -7.950 |
| GB63 | EU native | M | 53.651 | -0.111 |
| GB670 | EU native | M | 53.694 | -0.761 |
| LI85 | EU native | M | 55.349 | 21.483 |
| GB60 | EU native | M | 56.458 | -3.051 |
| DK609 | EU native | M | 57.092 | 9.051 |
| RU306 | EU native | M | 57.183 | 39.417 |
| RU169 | EU native | M | 59.894 | 30.264 |
| TN174 | EU native | M | 33.883 | 10.117 |
| GRkos1 | EU native | M | 36.891 | 27.285 |
| ES881 | EU native | M | 37.383 | -5.996 |
| ES681 | EU native | M | 37.981 | -0.685 |
| ITsard5 | EU native | M | 39.019 | 8.377 |
| ITsard768 | EU native | M | 40.698 | 9.724 |
| ITsard13 | EU native | M | 40.926 | 8.797 |
| ITsard15 | EU native | M | 41.013 | 8.879 |
| ES960 | EU native | M | 41.276 | 1.987 |
| IT1008 | EU native | M | 43.789 | 7.607 |
| ITric2 | EU native | M | 44.001 | 12.651 |
| IT753 | EU native | M | 44.002 | 12.652 |
| IT769 | EU native | M | 44.167 | 11.332 |
| IT882 | EU native | M | 44.452 | 11.192 |
| IT883 | EU native | M | 44.468 | 12.283 |
| IT756 | EU native | M | 44.614 | 12.244 |
| CR878 | EU native | M | 44.858 | 15.604 |
| ITpo1 | EU native | M | 44.935 | 12.445 |
| SL799 | EU native | M | 45.550 | 13.730 |
| SL800 | EU native | M | 45.938 | 14.549 |
| ITpo2 | EU native | M | 45.967 | 11.596 |
| SL961 | EU native | M | 46.306 | 15.608 |
| SL963 | EU native | M | 46.413 | 16.110 |
| SL962 | EU native | M | 46.415 | 16.137 |
| SW801 | EU native | M | 47.219 | 8.693 |
| HU885 | EU native | M | 47.477 | 19.042 |
| HU887 | EU native | M | 47.650 | 19.071 |
| UK959 | EU native | M | 50.794 | -4.555 |
| GB682 | EU native | M | 51.543 | 0.166 |
| DK20 | EU native | M | 55.398 | 8.453 |
| DK1016 | EU native | M | 56.145 | 10.089 |
| DK1019 | EU native | M | 56.145 | 10.089 |
| DK1020 | EU native | M | 56.145 | 10.089 |
| DK1026 | EU native | M | 56.145 | 10.089 |
| DK1011 | EU native | M | 56.146 | 10.089 |
